# Circulating GATA2 mRNA is decreased among women destined to develop preeclampsia and may be of endothelial origin

**DOI:** 10.1038/s41598-018-36645-0

**Published:** 2019-01-18

**Authors:** Carole-Anne Whigham, Teresa M. MacDonald, Susan P. Walker, Natasha Pritchard, Natalie J. Hannan, Ping Cannon, Tuong Vi Nguyen, Roxanne Hastie, Stephen Tong, Tu’uhevaha J. Kaitu’u-Lino

**Affiliations:** 1 Translational Obstetrics Group, The Department of Obstetrics and Gynaecology, Mercy hospital for Women, University of Melbourne, 163 Studley Road, Heidelberg, 3084 Victoria, Australia; 2 Mercy Perinatal, Mercy Hospital for Women, Victoria, Australia

## Abstract

Preeclampsia is a pregnancy complication associated with elevated placental secretion of anti-angiogenic factors, maternal endothelial dysfunction and organ injury. GATA2 is a transcription factor expressed in the endothelium which regulates vascular homeostasis by controlling transcription of genes and microRNAs, including endothelial miR126. We assessed GATA2 and miR126 in preeclampsia. Whole blood circulating *GATA2* mRNA and miR126 expression were significantly decreased in women with established early-onset preeclampsia compared to gestation-matched controls (p = 0.002, p < 0.0001, respectively). Using case-control groups selected from a large prospective cohort, whole blood circulating *GATA2* mRNA at both 28 and 36 weeks’ gestation was significantly reduced prior to the clinical diagnosis of preeclampsia (p = 0.012, p = 0.015 respectively). There were no differences in *GATA2* mRNA or protein expression in preeclamptic placentas compared to controls, suggesting the placenta is an unlikely source. Inducing endothelial dysfunction *in vitro* by administering either tumour necrosis factor-α or placenta-conditioned media to endothelial cells, significantly reduced *GATA2* mRNA expression (p < 0.0001), suggesting the reduced levels of circulating *GATA2* mRNA may be of endothelial origin. Circulating *GATA2* mRNA is decreased in women with established preeclampsia and decreased up to 12 weeks preceding onset of disease. Circulating mRNAs of endothelial origin may be a novel source of biomarker discovery for preeclampsia.

## Introduction

Preeclampsia is a multi-system disease that complicates 3–8% of pregnancies worldwide each year and 10–15% of direct maternal deaths are attributable to preeclampsia or eclampsia^1^. It is characterized by maternal hypertension after 20 weeks’ gestation and evidence of end-organ injury in the mother, such as injury to the kidneys (proteinuria), liver (raised transaminases) and neurological system (visual disturbances or an eclamptic seizure), as well as placental insufficiency. It is associated with 3 million premature births annually^2^.

Early-onset preeclampsia is defined as occurring before 34 weeks’ gestation whereas late-onset occurs after 34 weeks’ gestation. The origin of early-onset preeclampsia most likely lies in the poorly perfused placenta secreting elevated levels of placental factors such as anti-angiogenic molecules and inflammatory cytokines^3^. These cause widespread endothelial cell dysfunction^4^ and vascular injury leading to the end organ damage seen in clinical disease. Although many aspects of the pathophysiology of early- and late-onset preeclampsia overlap, it is thought that the primary mechanism of disease in late-onset preeclampsia is maternal endothelial susceptibilities, rather than poor placentation^5^. Endothelial dysfunction is present in both categories however in late-onset preeclampsia there are maternal factors that drive the endothelial dysfunction while maintaining normal placentation. As a result, many of the attempts at identifying differentially expressed genes for late-onset preeclampsia using placental proteins are not successful.

Tests to identify mothers at high risk of developing preeclampsia have been avidly sought as they could be used to triage for more effective care, and to direct potential preventative therapies. Recently, a first trimester test has been identified that combines, circulating placental growth factor (PIGF), uterine artery Doppler flow measured by ultrasound (increased resistance is associated with early placental dysfunction) and the mother’s mean arterial pressure^6^. This test is sensitive in predicting early onset preeclampsia and identifying mothers who screen high risk in this test may allow obstetricians to intervene with increased monitoring to avoid the consequences of preeclampsia^7^. However, this test is not good at identifying those destined to develop preeclampsia occurring near term gestation (i.e. late onset preeclampsia) and therefore still misses most cases of the disease^7^.

Numerous differentially expressed placental proteins have been identified in the circulation of those who have established preeclampsia^8,9^. These include soluble fms-like tyrosine kinase-1 (sFlt-1), soluble endoglin (sEng)^10,11^, pregnancy associated plasma protein-A (PAPP-A)^6^, PIGF^6^, and others. Notably, they are almost all placentally derived, and even when examined in combination, have not resulted in a predictive blood biomarker test that is useful for clinical practice. However, previous work has shown that free circulating mRNA may be a reliable and a novel source of biomarker discovery^12^. It may be possible that combining differentially expressed mRNA of both placental and endothelial origin is a promising approach to develop more accurate predictive tests for preeclampsia. To date, there have been very few differentially expressed mRNA molecules of endothelial origin identified as predictive markers of preeclampsia.

*GATA2* is a nuclear transcription factor expressed in the endothelium that regulates endothelial cell function and angiogenesis. It controls the expression of genes that are important in maintaining vascular function, such as platelet/endothelial adhesion molecule (PECAM-1)^13^, Flk-1^14^, endothelin-1 (ET-1)^15^ and vascular cell adhesion molecule-1 (VCAM-1)^16^. Furthermore, GATA2 has recently been shown to regulate endothelial, proangiogenic microRNA 126 (miR126) and antiangiogenic microRNA 221^17^. MiR126 has been shown to regulate vascular endothelial growth factor A (VEGF-A)^18^, which has specific endothelial cell functions such as promotion of angiogenesis, and growth of endothelial cells.

In view of the vital role that *GATA2, miR 126 and miR 221* have in maintaining endothelial cell function, combined with the knowledge that late-onset preeclampsia is a disease of dysfunctional endothelium, we sought to examine whether circulating levels are differentially expressed in preeclampsia. mRNAs are shed from various tissues into the maternal circulation where they can be readily quantified^19–22^. Therefore, we took the novel approach of measuring circulating mRNA levels of GATA2 in whole blood. We utilized bloods collected from women with established disease and also from a large prospective cohort study of samples collected both at 28 weeks’ and 36 weeks’ gestation from women preceding disease diagnosis. We also performed observational and functional studies to determine whether the circulating *GATA2* mRNA may be of endothelial or placental origin.

## Results

### Circulating *GATA2* mRNA is reduced among women with preeclampsia, and those destined to later develop late-onset preeclampsia

**Figure 1:**
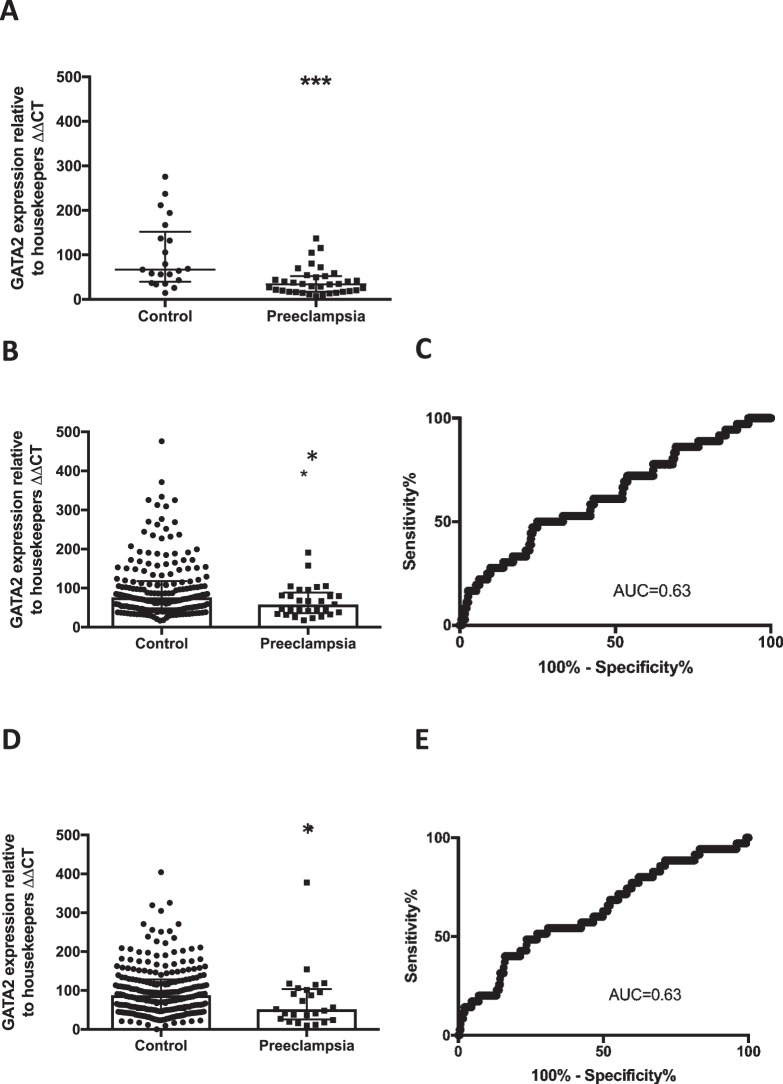
*GATA2* mRNA expression is reduced in maternal whole blood in patients with established preeclampsia as well as in those destined to develop the disease. (**A**) *GATA2* mRNA expression was significantly reduced (p = 0.002) in patients with severe early onset preeclampsia (n = 34) compared to gestation matched preterm controls (n = 21) with no preeclampsia. (Data displayed as median with interquartile range.) (**B**) *GATA2* was significantly reduced at 36 weeks in the FLAG cohort (p = 0.015) in patients destined to develop preeclampsia (n = 39) compared to those who did not go onto develop the disease (n = 205). (Data displayed as median with interquartile range.) (**C**) ROC curve at 36 weeks shows area under the curve (AUC) 0.63. (**D**) *GATA2* mRNA was also significantly reduced in the FLAG cohort at 28 weeks (p = 0.012) in patients destined to develop preeclampsia (n = 39) compared to controls (n = 248). (Data displayed as median with interquartile range.) (**E**), ROC curve shows AUC 0.63 at 28 weeks. *p < 0.05, **p < 0.01, ***p < 0.001, ****p < 0.0001.

We initially measured circulating *GATA2* mRNA in whole blood among 34 women with established early onset preeclampsia (delivered <34 weeks’ gestation), compared with 21 normotensive healthy pregnancies who subsequently delivered at term. Bloods were collected at the same gestational age from both cohorts and clinical characteristics of the participants are shown in Supplementary Table 1. Circulating *GATA2* mRNA concentrations were significantly reduced, by ~35% among participants with preeclampsia, compared to controls (p = 0.002, Fig. 1A).

Given circulating *GATA2* mRNA levels were changed among women with established preeclampsia, we next examined whether levels were differentially expressed prior to the development of late-onset disease. The FLAG (Fetal Longitudinal Assessment of Growth) study was a large prospective cohort study where bloods were collected from ~2000 participants at 28 and 36 weeks’ gestation. We performed a nested case-control study where we measured circulating *GATA2* mRNA levels at 28 weeks in participants who went on to develop late-onset preeclampsia and controls (maternal characteristics summarised in supplementary table 2) and similar groups at 36 weeks’ gestation (the maternal characteristics and clinical outcomes for these participants are summarised in Table 1, demographics and pregnancy outcome data in supplementary table 3). All these cases were selected from among the first 1000 participants of FLAG.

**Table 1:** Demographics of patients analysed at 36 weeks prior to diagnosis of preeclampsia compared with control.

		Controls (n = 193)	Preeclampsia (n = 37)	*P*
**Age** (mean)		33.0	32.3	0.34
**GDM**		22% (42)	22% (8)	0.83
**Smoker**		6% (12)	22% (8)	>0.99
**Parity**	Primip	63% (121)	72% (26)	0.52
**Gestational age at delivery** (weeks/mean)		39.5	39.11	0.08
**Birthweight** (Mean)		3464	3297	0.04
**Mode of delivery**	Normal Vaginal	45% (87)	30% (11)	
	Instrumental	19% (37)	16% (6)	
	Caesarean Section	36% (69)	54% (20)	
**BMI** (mean)		24	30	<0.0001

No significant difference in age, gestational diabetes status, parity, gestational age at delivery. A significant difference between the two groups can be seen in birthweight and BMI.

Circulating *GATA2* mRNA was significantly reduced in women destined to develop preeclampsia at 36 weeks’ gestation, compared to controls (Fig. 1B, p = 0.015), with an area under the receiver operating characteristic (ROC) curve (AUC) of 0.63 (Fig. 1C). Notably, circulating *GATA2* mRNA levels at 28 weeks’ gestation (as far as 10–12 weeks preceding diagnosis for most patients) were also significantly decreased among those destined to develop preeclampsia near term (the diagnosis of the disease was made after 36 weeks’ gestation) compared to controls. (Fig. 1D, p = 0.012), with an AUC of 0.63 (Fig. 1E). Therefore, it appears that preeclampsia is associated with decreased circulating *GATA2* mRNA levels preceding clinical diagnosis.

Given the BMI between our controls and preeclamptic cohorts was significantly different we assessed whether there was any correlation between BMI and GATA2 mRNA expression, however found no significant relationship (data not shown) suggesting the change in GATA2 mRNA expression between groups was independent of BMI.

### Circulating miR126 expression is reduced in established early onset preeclampsia

**Figure 2:**
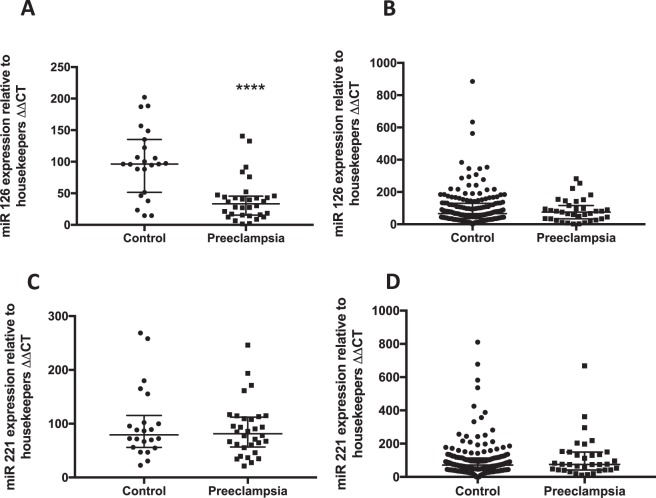
miR126 is decreased in the circulation of patients with established preeclampsia but is unchanged at 36 weeks before the onset of the disease; miR 221 is unchanged in established PE and at 36 weeks in those destined to develop PE. (**A**) *miR126* expression was significantly reduced (p < 0.0001) in patients with severe early onset preeclampsia (n = 34) compared to gestation matched preterm controls with no preeclampsia (n = 21). (**B**) *miR126* was unchanged in the FLAG cohort at 36 weeks (p = 0.74) in patients destined to develop preeclampsia (n = 39) compared to those who did not go onto develop the disease (n = 205). (**C**) *miR 221* expression was unchanged (p = 0.76) in patients with severe early onset preeclampsia compared to gestation matched preterm controls with no preeclampsia. (**D**) *miR221* was unchanged at 36 weeks (p = 0.57) in patients destined to develop preeclampsia compared to those who did not go onto develop the disease. *p < 0.05, **p < 0.01, ***p < 0.001, ****p < 0.0001. (All data displayed as median with interquartile range.)

It has been shown previously that *GATA2* regulates expression of pro-angiogenic miR126 and anti-angiogenic miR221^17^. Thus, given our data demonstrates significantly reduced circulating *GATA2* mRNA, we set out to measure expression of these circulating miRs in the same samples. In patients with established, severe early onset preeclampsia we found significantly decreased miR126 expression compared with controls (Fig. 2A, p < 0.0001). However, we found no significant change in miR126 expression at 36 weeks before the onset of preeclampsia (Fig. 2B, p = 0.74).

We also found no significant differences in miR 221 expression in mothers with established preeclampsia (Fig. 2C, p = 0.76) or at 36 weeks in those destined to develop preeclampsia (Fig. 2D, p = 0.57). Thus, we conclude that whilst pro-angiogenic miR126 is significantly reduced in established preterm disease, it is not altered preceding term disease.

### GATA2 expression is not altered in preeclamptic placentas

**Figure 3:**
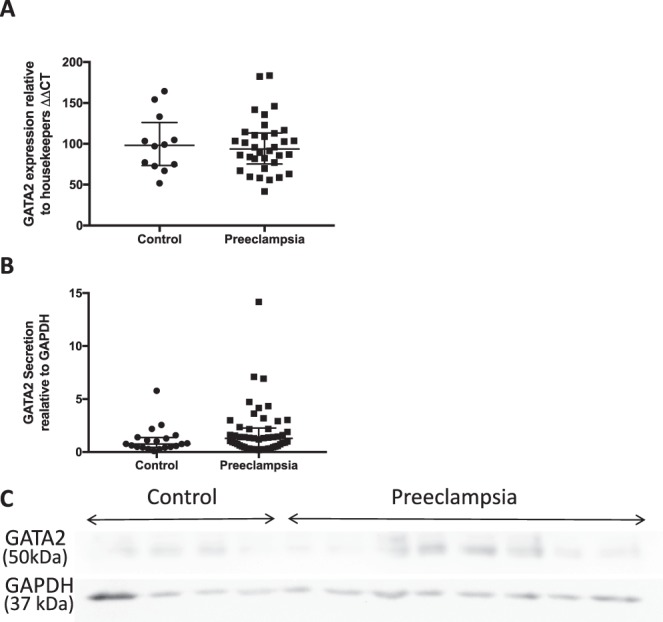
GATA2 mRNA and protein are not altered in preeclamptic placentas GATA2 mRNA and protein expression were assessed in placentas collected from patients with severe early onset (<34 weeks’ gestation) preeclampsia compared to pre-term control placentas. (**A**) There was no difference in *GATA2* mRNA expression (p = 0.94) in placentas from patients with severe early onset preeclampsia (n = 34) compared with control (n = 12). (Data displayed as median with interquartile range) (**B**) Similarly, there was no significant difference in GATA2 protein expression (p = 0.17) in preeclamptic placentas (n = 53) compared with control (n = 17) as measured by Western Blot analysis. (Data displayed as median with interquartile range.) (**C**). *p < 0.05, **p < 0.01, ***p < 0.001, ****p < 0.0001.

Most proposed circulating biomarkers of preeclampsia are of placental origin. Therefore, we set out to establish whether *GATA2* mRNA is altered in placentas collected from women with established preeclampsia. We measured *GATA2* mRNA in 34 placentas from women with preterm preeclampsia, compared to 12 controls (from preterm deliveries where the women remained normotensive and did not develop preeclampsia). The baseline clinical characteristics of participants from which the mRNA from placentas were obtained are shown in Supplementary Table 4. Interestingly, *GATA2* mRNA expression was not significantly different in preeclamptic placentas compared to controls (Fig. 3A, p = 0.94,). As GATA2 is a transcription factor we next assessed protein expression within the placenta. We increased the cohort size, including 53 placentas from women with preterm preeclampsia and 17 normotensive controls (Patient Demographics Supplementary Table 5) and similarly found no significant change in GATA2 protein expression (Fig. 3B,C, p = 0.17). Therefore, we conclude that cellular mRNA and protein expression of GATA2 is not altered in preeclamptic placentas and suggest that the altered circulating *GATA2* mRNA observed in established disease and preceding disease diagnosis may not be of placental origin. We note however that the possibility of differential export from preeclamptic placenta remains to be tested.

### *GATA2* is decreased in HUVECs exposed to cytotrophoblast conditioned media and TNFα

Another possible source of circulating *GATA2* is the endothelial cells. Given endothelial tissues from women with preeclampsia are not readily accessible, we assessed whether we could modulate *GATA2* mRNA expression in functional models of endothelial dysfunction using primary HUVECs as a model.

We initially used a model of endothelial dysfunction induced by placental factors^23^. Our previous work reports that trophoblast conditioned media contains both the anti-angiogenic molecules sFlt-1 and soluble endoglin^24^, and is also likely to contain other vaso-active molecules that induce endothelial dysfunction in primary HUVECs. Thus, here we obtained conditioned media from freshly isolated cytotrophoblast cells, before exposing primary HUVECs (human umbilical vein endothelial cells, used to model the endothelium) to this media, or control media^23^.

We also chose to include a second model of inflammatory-mediated endothelial dysfunction by treating HUVECs with tumour necrosis factor-α (TNFα). TNFα is a pro-inflammatory cytokine that is elevated in the circulation of women with preeclampsia^25^. In addition it has been proposed that oxidative stress from the preeclamptic placenta may release cytokines such as TNFα into the maternal circulation inducing systemic endothelial dysfunction^26^.

**Figure 4:**
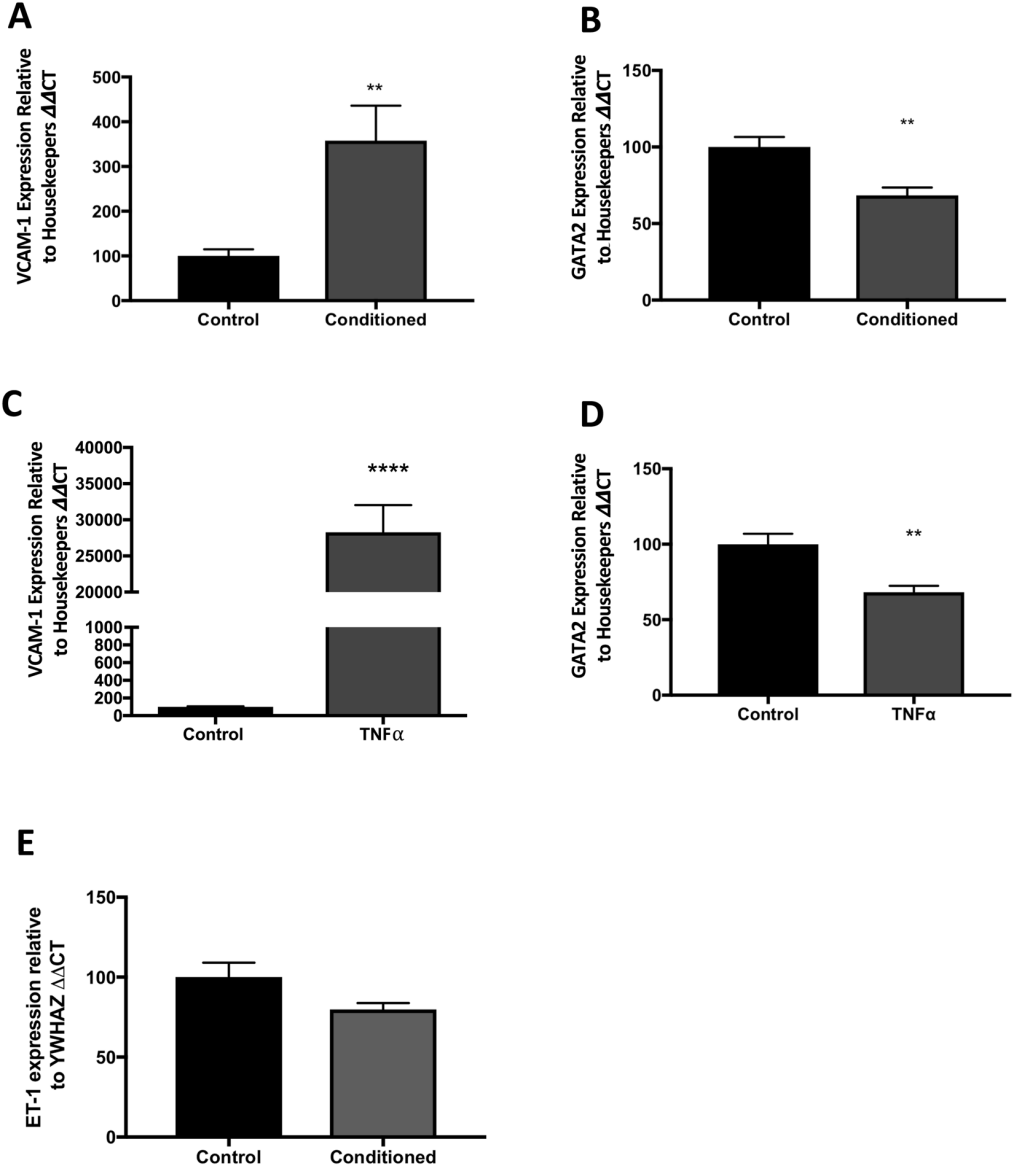
Endothelial *GATA2* mRNA is significantly decreased by placental factors in primary HUVECs. After exposure of primary HUVECs to cytotrophoblast conditioned media there was significantly (**A**) increased *VCAM-1* (p = 0.005) (data displayed as mean with standard error of the mean,) and (**B**) decreased *GATA2* (p = 0.002) (data displayed as mean with standard error of the mean.) When HUVECS were treated with TNFα we found (**C**) significantly increased *VCAM-1* (p < 0.0001*)* (data displayed as mean with standard error of the mean) along with (**D**) significantly decreased *GATA2* (p = 0.002). (Data displayed as median with interquartile range.) (**E**) *ET-1* was unchanged when cells are treated with trophoblast conditioned media (p = 0.06). Experiments were repeated a minimum of n = 3 times, with each ‘n’ representing a separate primary HUVEC isolation and including experimental triplicates. *p < 0.05, **p < 0.01, ***p < 0.001, ****p < 0.0001.

The administration of either cytotrophoblast conditioned media, or TNF**α** to HUVECs significantly increased endothelial dysfunction marker, *VCAM-1* expression (Fig. 4A,C, p < 0.0001). *ET-1* was unchanged in cells treated with conditioned media (Fig. 4E, p = 0.06) Simultaneously, cytotrophoblast conditioned media significantly reduced *GATA2* expression in HUVECs (Fig. 4B, p = 0.002). Similarly, TNF**α** also significantly reduced *GATA2* mRNA expression in primary HUVECs (Fig. 4D, p = 0.002). Together, this data provides evidence that endothelial *GATA2* mRNA expression is reduced by inducing endothelial dysfunction, using either media rich in placental factors, or TNFα. It provides functional evidence to suggest that similar changes may result in the reduced circulating GATA2 observed in the circulation of women preceding, or with established preeclampsia.

### *GATA2* is unchanged in peripheral blood monocytes (PBMCs) and granulocytes exposed to cytotrophoblast conditioned media

Given leukocytes are an important source of circulating nucleic acids in whole blood^27^ we also examined whether PBMCs and granulocytes may contribute to the reduced circulating *GATA2* mRNA expression. Similar to our endothelial experiments, we isolated white blood cells from pregnant women and incubated them with (or without) cytotrophoblast conditioned media to determine whether placental factors would alter their *GATA2* mRNA expression. In contrast with our findings seen in endothelial cells (HUVECs), there was no significant difference in *GATA2* mRNA expression between cells treated with conditioned cytotrophoblast media vs cells treated with control media (Supplementary Figure 1 p = 0.97). Therefore, we conclude that placental factors do not alter *GATA2* mRNA expression in maternal PBMCs and granulocytes.

### Resveratrol rescues TNFα-induced GATA2 mRNA repression in endothelial cells

**Figure 5:**
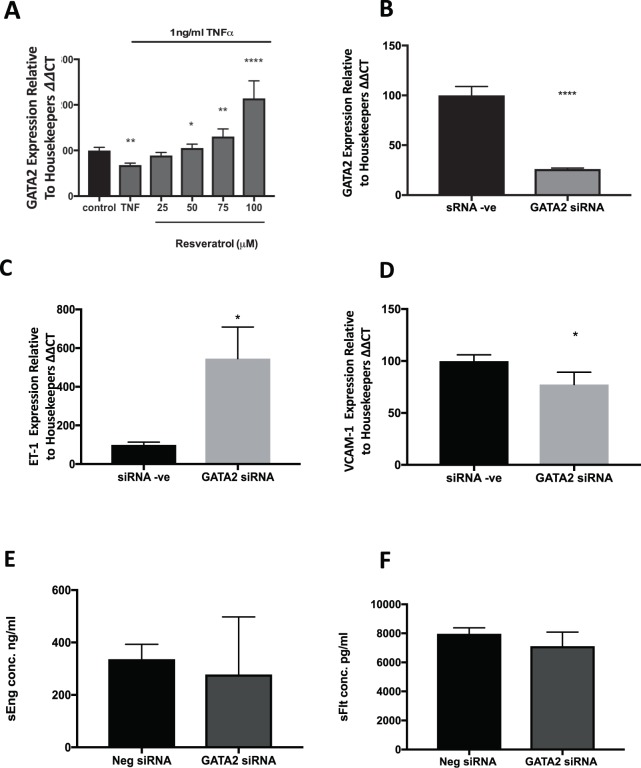
Resveratrol dose dependently reverses *GATA2* down regulation. (**A**) *GATA2* mRNA is significantly reduced when HUVECS are treated with TNFα, and this effect is reversed in a dose-dependent manner following treatment with resveratrol. Silencing *GATA2* in primary HUVECs. To determine the effect of reduced *GATA2* on anti-angiogenic factor secretion, *GATA2* was silenced in primary HUVECs using siRNA. (**B**) siRNA targeting *GATA2* significantly reduced its mRNA expression (p < 0.0001, data displayed as mean with standard error of the mean). (**C**) There was a significant increase in *ET-1* when GATA2 was silenced (data displayed as mean with standard error of the mean); (**D**) *VCAM-1* was significantly reduced (data displayed as mean with standard error of the mean). There was no significant change in secretion of (**E**) sEng (data displayed as median with interquartile range) or (**F**) sFlt-1 in these same cells (data displayed as median with interquartile range). Experiments were repeated a minimum of n = 3 times, with each ‘n’ representing a separate primary HUVEC isolation and including experimental triplicates. *p < 0.05, **p < 0.01, ***p < 0.001, ****p < 0.0001.

We next investigated if there was a treatment that could rescue the decrease in *GATA2* expression seen with endothelial dysfunction. Our group has previously published studies on a number of therapeutics that rescue endothelial dysfunction^23,28–31^. As such we wondered if any of these therapeutics might modulate *GATA2* mRNA expression, which we found was reduced in endothelial dysfunction. We repeated the model of endothelial dysfunction *in vitro* by administering TNFα to HUVECs in the presence or absence of the anti-oxidant resveratrol^30^, anti-cholesterol agent pravastatin^23^, proton pump inhibitor esomeprazole^31^ or anti-diabetic drug metformin^29^. Resveratrol significantly increased *GATA2* mRNA expression in a dose dependent manner (Fig. 5A), however we observed no significant change in *GATA2* mRNA expression following treatment with the other drugs (Supplementary Figure 2). Thus, we conclude, that although we have previously demonstrated positive effects of these agents in models of endothelial dysfunction^23,29–31^, only resveratrol can induce *GATA2* mRNA expression.

### Silencing *GATA2* increases ET-1 expression but does not affect sFlt-1 or sEng production in HUVECs

We next investigated the effects of silencing GATA2 on markers of endothelial dysfunction, *VCAM-1* and *ET-1*. Using siRNA targeting *GATA2*, we were able to decrease *GATA2* expression by 74% in primary HUVECs (Fig. 5B). We demonstrated a significant increase in *ET-1* (Fig. 5C, p = 0.02), however, surprisingly, found *VCAM-1* (Fig. 5D, p = 0.02) was reduced when *GATA2* was silenced.

sFlt-1 and soluble endoglin (sEng) are antiangiogenic factors that are significantly increased in the circulation of patients with preeclampsia and are likely to play a major role in inducing endothelial dysfunction in the condition^10,11^. While the placenta is likely to be a major source of these factors, a second important source is the endothelium itself^32^. Given GATA2 is a transcription factor, we examined whether it may be involved in the upstream regulation of sFlt-1 or soluble endoglin (sEng) in endothelial cells by also measuring sFlt-1 and sEng secretion from primary HUVECs where GATA2 was silenced. siGATA2 administered to HUVECs did not affect sEng (Fig. 5E) or sFlt-1 (Fig. 5F) secretion, suggesting it does not regulate either of these anti-angiogenic factors in endothelial cells.

## Discussion

In this report, we show that circulating mRNA levels of the endothelial transcription factor *GATA2* are significantly decreased in preeclampsia; not only in established early-onset disease but also at 28 and 36 weeks’ gestation, before the development of late-onset disease. Further, we have undertaken a combination of functional studies as well as examining expression in the placenta, and PBMCs/platelets, to suggest that the depressed circulating *GATA2* mRNA detected may be of endothelial cell origin.

Free circulating mRNA is a rich source of potential biomarkers^10,11^ and these are currently being investigated for use in cancer diagnosis and prognosis, as well as other pregnancy related complications such as fetal growth restriction^33^. There have been several studies looking at circulating differentially produced proteins in preeclampsia versus control but comparatively far less investigation into measuring differentially expressed circulating mRNAs for this same purpose. The FLAG cohort is a large prospective cohort where 3.9% of patients developed preeclampsia following blood sampling, and therefore is an ideal cohort to investigate differentially expressed mRNA before the onset of disease. In contrast to many studies, our blood samples for mRNA and miRNA assessment were collected and extracted from PaxGene tubes, a system that is optimised specifically to maintain RNA and miRNA quality for measurement of circulating nucleic acids^34^.

*GATA2* is known to play a vital role in endothelial cell function and development. Given that expression of *GATA2* is reduced in diseases of endothelial cell dysfunction outside of pregnancy^17^ we wondered if the same may be true of the dysfunctional endothelium present in preeclampsia. Although it is widely recognised that maternal endothelial dysfunction is a major step in the development of preeclampsia, there has been no previous studies of circulating *GATA2* mRNA in the disease. Indeed, we found significantly decreased circulating *GATA2* mRNA in both established early-onset preeclampsia and preceding diagnosis of late-onset preeclampsia. Unfortunately, we did not have access to mRNA from established late-onset disease, however our data at both 28 and 36 weeks’ gestation strongly suggests that *GATA2* mRNA would also be significantly decreased in such samples. These findings need further validation in other data sets, and we endeavour to carry out validation in the second thousand FLAG samples in the future.

Although a previous study demonstrated a relationship between GATA2 and miR126 and miR221, we were unable to find any changes in miR221in our samples. In contrast, miR126 was significantly decreased in established disease but no change was identified preceding disease onset. We believe that this lack of change reflects disease progression. Although our circulating data suggests GATA2 is reduced early in the disease process (up to 10–12 weeks before disease onset) it appears circulating miR126 levels are not reduced until disease onset. This is in keeping with previous studies that demonstrate changes in miR126 in established endothelial cell dysfunction^17^. We are unaware of any studies that have found changes in miR126 preceding endothelial disease. Whilst previous studies have shown that GATA2 regulates expression of miR126^17^, we note that our study does not demonstrate a causal relationship between the reduced circulating GATA2 and reduced circulating miR126 in established disease.

It has been shown that *GATA2* regulates markers of endothelial cell dysfunction including VCAM-1, ET-1 and nitric oxide synthase^17^. We have confirmed that this is also likely in preeclampsia in our functional experiments, demonstrating that placental factors or TNFα significantly reduced endothelial *GATA2* coincident with increased VCAM-1 expression. However, the exact placental factors that are responsible for significantly reducing endothelial GATA2 remains to be elucidated. We acknowledge that HUVECs do not represent a perfect model but are used here as they are easily accessible and have been used recurrently in the field^10,11,29^. We further found that, although inducing endothelial cell dysfunction with TNFα or placental factors were associated with rising VCAM-1, when *GATA2* was silenced in healthy cells, VCAM-1 was down regulated suggesting *GATA2* may not be causal of endothelial dysfunction.

Interestingly, we demonstrated that resveratrol rescued the TNFα-induced down regulation of *GATA2* mRNA expression in our model of endothelial dysfunction. This finding builds on our recent work showing resveratrol dose dependently reduced sFlt-1 and sEng secretion from primary cytotrophoblasts^30^ and endothelial cells, although our studies reported here suggest the reduced secretion of these molecules are not mediated through the increase in GATA2 expression. Together this data suggests that resveratrol has potential as a therapy, which could treat not only placental dysfunction in preeclampsia but also maternal endothelial dysfunction. We further suggest that the decrease in endothelial dysfunction induced by resveratrol may be via a *GATA2* pathway. Some caution is however required before translating this information to human studies given a recent primate study suggesting maternal resveratrol administration may enlarge the fetal pancreas. Small numbers were used in that study and the implications of an enlarged pancreas has not yet been determined, therefore human trials may still be considered in the future^35^.

### Perspectives

This study provides the first data to demonstrate that circulating *GATA2* mRNA is significantly reduced, not only in established preeclampsia, but also as early as 10–12 weeks preceding the clinical diagnosis of the disease. Our functional studies have indicated that free circulating *GATA2* mRNA may be of endothelial cell origin and potentially the reduced levels are associated with endothelial cell dysfunction. We propose that *GATA2* mRNA may be a new candidate for inclusion in a multi-marker biomarker test to detect those who will go on to develop preeclampsia. Using a maternally derived biomarker to predict those at risk of preeclampsia is a novel concept given most suggested biomarkers for preeclampsia (e.g. PlGF, sFlt-1) are of placental origin. Free circulating *GATA2* mRNA level could be combined with placental biomarkers or other endothelial biomarkers to improve the sensitivity and specificity in predicting preeclampsia.

## Materials and Methods

### Established Preeclampsia- cases and controls: Blood Collection

Women presenting to the Mercy Hospital for Women, a tertiary maternity hospital in Melbourne with approximately 6,000 births annually, gave informed written consent for placental tissue and/or blood collection. We obtained 21 blood samples from women with preterm pregnancies not complicated by preeclampsia, and 34 samples from women with severe early-onset preeclampsia. Preeclamptics were diagnosed in accordance with the American College of Obstetrician and Gynecologists (ACOG) guidelines 2013^36^. All samples were obtained from cases of early-onset preeclampsia (<34 weeks’ gestation). Preterm controls were selected from women with pre-term rupture of membranes, placenta praevia or antepartum haemorrhage and did not have any evidence of infection (histopathological examination of the placentas), hypertensive disease or maternal co-morbidities. Whole blood was collected in a PAXgene RNA tube at the time of consent. Human Ethics approval was obtained for this study from the Mercy Health Human Research Ethics Committee (R11/34). All methods were performed in accordance with the relevant guidelines and regulations. All patients delivered by caesarean section. Patient characteristics are shown in Supplementary Table 1.

### FLAG Study design overview

This analysis is part of the Fetal Longitudinal Assessment of Growth (FLAG) study carried out at the Mercy Hospital for Women. The FLAG study, designed to identify biomarkers to detect small for gestational age (SGA) fetuses, included prospective collection of 2,015 blood samples from pregnant women at 28 and 36 weeks’ gestation (collected Feb 2015-May 2016). 3.9% of patients developed preeclampsia.

We performed a case-control study using blood samples chosen from the first 1,000 FLAG participants. We compared the 36 and 28 week *GATA2* mRNA values from women who went on to develop preeclampsia after 36 weeks, to the analyte levels in control bloods. We chose controls at a 2:1 ratio with those participants who went on to develop FGR; our analysis for preeclampsia was a sub-study in which we kept the same controls. At 36 weeks’ gestation, we analysed 205 controls. Twelve were excluded due to technical processing errors (e.g. poor PCR reads for either the housekeeping genes, target genes or both). In the patients destined to develop preeclampsia, 39 were analysed but 2 excluded due to technical processing errors. Seven of these patients had a pregnancy complicated by fetal growth restriction as well as preeclampsia. At 28 weeks, 248 controls were run, 1 excluded; 39 from those with preeclampsia run with 3 excluded.

This study was approved by the Mercy Health Research Ethics Committee (Ethics Approval Number R14/12, R11/34) and written informed consent was obtained from all participants. All methods were performed in accordance with the relevant guidelines and regulations.

### FLAG recruitment

Women were screened for eligibility and invited to participate at their oral glucose tolerance test, offered to all women around 28 weeks’ gestation to diagnose gestational diabetes mellitis (part of routine care). English-speaking women aged over 18 years, carrying a well-dated singleton pregnancy with normal mid-trimester morphology ultrasound were eligible to participate. Whole blood was collected in a PAXgene RNA tube at the time of enrollment, i.e. 27^+0^–29^+0^ weeks and at 35^+0^ to 37^+0^ weeks’ gestation inclusive.

### Outcomes and diagnostic criteria

Maternal characteristics and pregnancy outcomes were obtained by review of each participant’s medical record, investigation results and hospital database entry (Table 1 for 36 week sample demographics, Supplementary Table 3 for 36 week PE characteristics and Supplementary Table 2 for 28-week sample demographics).

Preeclampsia was diagnosed according to The American College of Obstetricians and Gynecologists’ Taskforce on Hypertension in Pregnancy definition^36^ new onset hypertension (blood pressure ≥140 mmHg systolic, or ≥90 mmHg diastolic on two occasions ≥ four hours apart after 20 weeks’ gestation); plus one of new-onset: proteinuria, thrombocytopaenia, renal insufficiency, impaired liver function, pulmonary oedema or cerebral symptoms.

### Established Preeclampsia- cases and controls: Tissue collection

We obtained preterm placentas from 12 pregnancies not complicated by preeclampsia and 34 placentas from those with established severe early-onset disease for RNA analysis. We utilised an expanded cohort of placentas for protein analysis. Seventeen control placentas and 53 from patients with preeclampsia were analysed for protein secretion. All samples were collected from patients delivering at <34 weeks’ gestation. Patient characteristics are shown in Supplementary Table 4 (mRNA) and 5 (protein).

Placental tissue was obtained immediately following delivery and samples taken from multiple sites according to CoLab recommendations^37^ Maternal and fetal surfaces were removed and the sample washed in sterile phosphate-buffered saline (PBS) before being placed in RNAlater (Life Technologies) and then stored at −80 °C.

### RNA extraction from PAXgene tubes

PAXgene blood RNA tubes were incubated for at least 2 hours at room temperature after blood collection, before storage at −80 °C. For extraction, tubes were thawed and then centrifuged for 10 mins at 3000–5000 *g*. Supernatant was discarded and the pellet resuspended in RNase free water before another centrifuge for 10 minutes at 3000–5000 *g*. The QIAcube (Qiagen, Valencia, CA) shaker was used as per manufacturer’s instructions to extract RNA. RNA was stored at −80 °C.

### RNA extraction

RNA was extracted from primary HUVECs, white blood cells or placental tissue according to manufacturer’s instructions using an RNeasy mini kit (Qiagen, Valencia, CA) and quantified using the Nanodrop ND 1000 spectrophotometer (NanoDrop technologies Inc, Wilmington, DE).

### Isolation of primary human umbilical vein endothelial cells

Human umbilical vein endothelial cells (HUVECs) were isolated as previously described^23^. Cells were cultured in M199 media (Life Technologies) containing 10% fetal calf serum (FCS, Life Technologies), 1% antibiotic-antimycotic (Life Technologies) and 1% endothelial cell growth factor (ECGS, Sigma) and 1% heparin and used between passages 2 to 4. Primary HUVECS were maintained in a humidified incubator with atmospheric O_2_ concentration, at 5% CO_2_.

### siRNA knockdown of GATA2

siRNA targeting *GATA2* was administered to primary HUVECs at 10 nM or a matching concentration of negative siRNA (Qiagen, Limburg, Netherlands) using lipofectamine RNAiMAX (Life Technologies). Primary HUVECs were cultured for 48 hours, with treatment of both *GATA2* siRNA and negative siRNA occurring after 24 hours of incubation.

Culture media was collected for analysis by ELISA and RNA collected for analysis by RT-PCR. Knockdown efficiency of *GATA2* siRNA in primary HUVECs was confirmed by qRT-PCR. (Supplementary Figure 3A). We initially confirmed no negative effect on viability using MTS (data not shown).

### cDNA and RT-PCR

RNA was converted to cDNA using Applied Biosystems high capacity cDNA reverse transcriptase kit (Life Technologies) or the miScript II RT kit (Qiagen), as per manufacturer guidelines.

Gene expression of *GATA2*, *VCAM-1, ET-1, YWHAZ, Topoisomerase-1, Cytochrome c1* and *GUSB, B2M* (Life Technologies) were quantified by real time PCR (qRT-PCR) on the CFX 384 (Bio-Rad, Hercules, CA) using FAM-labeled Taqman universal PCR master mix and its specific primer/probe set (Life Technologies) with the following run conditions: 50 °C for 2 minutes; 95 °C for 10 minutes, 95 °C for 15 seconds, 60 °C for 1 minute (40 cycles). *GUSB, YWHAZ and B2M* were used as housekeepers for PAXgene whole blood analyses. *YWHAZ* was used as a housekeeper for mRNA analyses on cells. *Topoisomerase-1* and *Cytochrome c1* were used as housekeepers for placental tissue. Data were analysed using the ΔΔCT method of analysis.

For microRNAs, the miScript SYBR green PCR kit was used. Housekeepers miR191, SNORD 44 and SNORD 48 were measured against miR126 and miR 221. The following conditions were used to carry out the PCR reaction-

Activation step: 95 °C for 15 mins, followed by 40 cycles of: 94 °C for 15 secs, 55 °C for 30 secs, 70 °C for 30 secs. Data were analysed using the ΔΔCT method of analysis.

### Isolation of white blood cells and platelets

Whole blood samples were collected in 10 ml EDTA tubes. Ammonium chloride red cell lysing solution was mixed with 1 ml blood from above tube to make up to 15mls. Every 1 ml of blood from each sample was treated the same way until all the sample contained lysing solution. Tubes were rocked at room temperature for 10 minutes until the liquid became clear red, then centrifuged at 4 °C for 10 minutes at 250*g. Supernatant was decanted and cells resuspended in 1 ml PBS/2% FCS. Multiple tubes from the same sample were combined. Cells remaining consisted of white cells- peripheral blood mononuclear cells and granulocytes. These were exposed to control media (DMEM, 1% heparin, 1% ECGS, 10% FCS) or cytotrophoblast conditioned media (DMEM, 1% antibiotic-antimycotic, 10% FCS that had been exposed to cytotrophoblasts) for 24 hours, maintained in a humidified incubator with atmospheric O_2_ at 5% CO_2._

### Western Blot Analysis

20 μg of placental lysates (n = 17 PT and n = 53 PE) were separated on 10% polyacrylamide gels with wet transfer to PVDF membranes (Millipore, Billerica, MA). Membranes were blotted overnight with an antibody targeting GATA2 (Rabbit anti-human GATA2, Sigma, 1:2000) or GAPDH (1:5000, Cell Signaling Technology, Danvers, MA, USA) and visualized using an enhanced chemiluminescence detection system (Santa Cruz Biotechnology, Santa Cruz, CA, USA) and ChemiDoc XRS (BioRad, Hercules, CA, USA). Relative densitometry was determined in all samples using Image Lab (BioRad).

### Endothelial dysfunction – cytotrophoblast conditioned media

Term placentas were collected from women having elective caesarean sections. Human cytotrophoblasts were isolated as previously described^24^. Primary cytotrophoblasts were cultured for 24 hours in DMEM high Glutamax (Life Technologies) containing 10% FCS and 1% antibiotic-antimycotic at 8% O_2_ and 5% CO_2_. Cells attached over 24 hours and the cytotrophoblast-conditioned media was then collected for treatment of primary HUVECs.

Primary HUVECs were seeded into 24-well plates for 24 hours before their media was removed and replaced at a 50:50 ratio with either HUVEC media (M199 containing 10% FCS, 1% anti-anti, 1% heparin, 1% ECGS) plus either cytotrophoblast conditioned media (i.e. media taken from trophoblasts in culture), or cytotrophoblast media (DMEM, 1% antibiotic-antimycotic, 10% FCS) that had not been exposed to cytotrophoblasts. HUVECs were exposed to the cytotrophoblast media for 24 hours before media was removed and cell lysates were subjected to RNA extraction and RT-PCR for *GATA2*, and *VCAM1*.

### Treatment of endothelial cells with Resveratrol, Pravastatin, Esomeprazole or Metformin

Endothelial dysfunction experiments were undertaken using primary HUVECs. HUVECs were isolated as above and pre-treated with 1 ng/ml TNFα (Sigma) for 2 hours, before increasing doses of resveratrol (0–100 μM), esomeprazole (0–100 μM), pravastatin (0–2000 μmol/L), metformin (0–5 mmol/L) or vehicle control (ethanol for resveratrol and esomeprazole, water for pravastatin and metformin) were added in the presence of TNFα for a further 24 hours. Cells were incubated at atmospheric O2, 5% CO2, 37 °C. At the cessation of the experiment, RNA was collected for qRT-PCR measurement of *GATA2, ET-1* and *VCAM-1*.

### Statistical analysis

Triplicate technical replicates were performed for *in vitro* experiments, with a minimum of three independent biological replicates performed for each *in vitro* study. Data was tested for normal distribution and statistically analysed as appropriate using t-tests if parametric or Mann-Whitney test if not. When three or more groups were compared, a 1-way ANOVA (for parametric data) or Kruskal-Wallis test (for non-parametric data) was used. All data is expressed as mean ± SEM or median with interquartile range for non-parametric data. P values < 0.05 were considered significant. Statistical analysis was performed using GraphPad Prism 7 software (GraphPad Software, La Jolla, CA).

The datasets generated during and/or analysed during the current study are available from the corresponding author on reasonable request.

## Electronic supplementary material

Supplementary Information
