# Supplementary material for: Circulating GATA2 mRNA is decreased among women destined to develop preeclampsia and may be of endothelial origin
Source: Sci Rep. 2019 Jan 18;9:235. doi: 10.1038/s41598-018-36645-0 (PMC6338784; doi:10.1038/s41598-018-36645-0)
Supplement: Supplementary file 1 — Supplementary Information [file 41598_2018_36645_MOESM1_ESM.docx]

**Circulating GATA2 mRNA is decreased among women destined to develop preeclampsia and may be of endothelial origin**

***Supplementary Datasets***

**Authors**

Carole-Anne Whigham^1,2^, Teresa M MacDonald^1,2^, Susan P Walker^1,2^, Natasha Pritchard^1,2^, Natalie J Hannan^1,2^, Ping Cannon^1,2^, Tuong Vi Nguyen^1,2^, Roxanne Hastie^1,2^, Stephen Tong^1,2,3^, Tu’uhevaha J Kaitu’u-Lino^1,2,3^

^1^ Translational Obstetrics Group, The Department of Obstetrics and Gynaecology, Mercy hospital for Women, University of Melbourne, 163 Studley Road, Heidelberg 3084, Victoria, Australia.

^2^ Mercy Perinatal, Mercy Hospital for Women, Victoria, Australia.

^3^ Indicates equal contribution

Corresponding Author:

Dr Carole-Anne Whigham

Mercy Hospital for Women

Dept. of Obstetrics and Gynaecology, University of Melbourne

163 Studley Road

Heidelberg, Vic 3084, Australia

Telephone: +613 8458 4377

Email: drcwhigham@gmail.com

| **Supplementary Table 1.**  **Maternal clinical characteristics - Bloods** | Controls (n=21) | Preeclamptic  (n=34) |
| --- | --- | --- |
| **Maternal Age** (years)  Median (IQR) | 30.9 (29.6-32.8) | 31.3 (28.2-33.8) |
| **Gestation at Delivery*****(weeks)  Median (IQR) | 39.0 (38.6 – 40.4) | 32.3 (29.7 – 38.9) |
| **Gestation at Blood Collection** (weeks)  Median (IQR) | 29.0 (28.0 - 30.3) | 29.3 (28.0-30.9) |
| **BMI** (kg/m^2^)*  Median (range) | 23 (21-28) | 25 (23-32) |
| **Parity** no. (%)  0  1  ≥2 | 11 (52)  9 (43)  1 (5) | 26 (76)  5 (15)  3 (9) |
| **SBP at Booking** (mmHg)  Median (IQR) | 108 (100-120) | 110 (100-120) |
| **SBP at Delivery** (mmHg)  Median (IQR) **** | 130 (127-132) | 160 (130-175) |
| **DBP at Delivery** (mmHg)  Median (IQR) **** | 75 (72-84) | 95 (80-103) |
| **Highest protein creatinine ratio (g/mmoL) §**  Median (IQR) | -- | 0.28 (0.09-0.68) |
| **Liver function abnormalities**  Number (%) | -- | 21 (62) |
| **Thrombocytopenia <150 (x10^9/L)**  Number (%) | -- | 13 (38) |

BMI = body mass index, SBP = systolic blood pressure and DBP = diastolic blood pressure. Mann-Whitney U tests used for comparison of medians. BMI data available for 30/34 preeclamptic women. SBP at booking available for 20/21 controls and 13/34 PE women. *p<0.05 ****p<0.0001 § Normal range <0.03

**Supplementary table 1: Demographics of patients with established severe early onset disease, blood samples.** No significant difference in maternal age or blood collection gestation. A significant difference can be seen in gestation at delivery, BMI, parity, birthweight and blood pressure readings.

**Supplementary Table 2**

|  |  | Controls (247) | Preeclampsia (37) | *P* |
| --- | --- | --- | --- | --- |
| **Age** (mean) |  | 32.2 | 32.3 | 0.87 |
| **GDM** |  | 0.4% (1) | 15% (6) | <0.0001 |
| **Smoker** |  | 7% (18) | 5% (2) | >0.999 |
| **Primip** |  | 65% (147) | 77% (28) | 0.07 |
| **Gestational age at delivery** (weeks/mean) |  | 39.4 | 39.36 | 0.58 |
| **Birthweight** (Mean) |  | 3450 | 2960 | <0.0001 |
| **Mode of delivery** | Normal Vaginal | 109 | 14 | 0.73 |
|  | Instrumental | 49 | 9 |  |
|  | Caesarean Section | 89 | 14 |  |
| **BMI** | (mean) | 26.9 | 29.9 | 0.01 |

**Supplementary Table 2: Demographics of patients analysed at 28 weeks prior to diagnosis of preeclampsia compared with control.** No significant difference in age, parity, gestational age at delivery. A significant difference between the two groups can be seen in birthweight, mode of delivery, gestational diabetes status and BMI.

**Supplementary Table 3**

|  |  | **Preeclampsia cases** |
| --- | --- | --- |
| **Systolic BP** (mean) | (mmHg) | 158 |
| **Diastolic BP** (mean) | (mmHg) | 97 |
| **Urine PCR** (mean) |  | 0.1 |
| **Platelets** (mean) | (Normal 150-400*10^9/L) | 166 |
| **Creatinine** (mean) | (Normal 44-80 umol/L) | 82 |
| **ALT** (mean) | (Normal 10-40 units/L) | 67 |
| **Onset of PE** | Antenatal | 19 |
|  | Intrapartum | 10 |
|  | Postnatal | 8 |
| **Symptomatic** |  | 8 |

**Supplementary Table 3: Clinical PE characteristics at 36 weeks of those destined to develop PE.** Table shows averages of parameters used to clinically diagnose preeclampsia. ALT=Alanine Transaminase, liver enzyme elevated in dysfunction. Urine PCR: Urine protein: creatinine ratio.

| **Supplementary Table 4.**  **Maternal clinical characteristics – Placental mRNA** | | |
| --- | --- | --- |
|  | Controls (n=12) | Preeclamptic  (n=34) |
| **Maternal Age** (years)  Median (IQR) | 31.3 (26.8-37.7) | 30.5 (27.6-32.4) |
| **Gestation at Delivery** (weeks)  Median (IQR) | 29.6 (28.2 – 30.4) | 30.0 (27.4 – 31.4) |
| **BMI** (kg/m^2^) ∞  Median (range) | 24.5 (23.7-31.3) | 27 (25-37) |
| **Parity** no. (%) *  0  1  ≥2 | 3 (25)  6 (50)  3 (25) | 24 (71)  6 (18)  4 (12) |
| **SBP at Booking** (mmHg)  Median (IQR) | 110 (110-120) | 120 (110-123) |
| **SBP at Delivery** (mmHg)  Median (IQR) **** | 120 (112-131) | 175 (160-182) |
| **DBP at Delivery** (mmHg)  Median (IQR) **** | 74 (70-80) | 100 (100-110) |
| **Birth weight** (g)  Median (IQR) | 1334 (1154-1552) | 1099 (851-1403) |
| **Highest protein creatinine ratio (g/mmoL) §**  Median (IQR) | -- | 0.27 (0.13-0.61) |
| **Liver function abnormalities**  Number (%) | -- | 20 (59) |
| **Thrombocytopenia <150 (x10^9/L)**  Number (%) | -- | 10 (29) |

BMI data available for 8/12 preterm controls and 28/34 preeclamptic women. SBP at booking available for 5/12 preterm controls and 15/34 preeclamptic women. *p<0.05 ****p<0.0001 § Normal range <0.03

**Supplementary table 4: Demographics of patients with established, severe early onset preeclampsia, mRNA placental tissue samples.** There was no significant difference seen in maternal age, gestation at delivery or BMI.

| **Supplementary Table 5.**  **Maternal clinical characteristics – Placental protein** | | |
| --- | --- | --- |
|  | Preterm controls (n=17) | Preeclamptic  (n=53) |
| **Maternal Age** (years)  Median (IQR) | 30.4 (25.6-36.4) | 31.1 (27.5-33.8) |
| **Gestation at Delivery** (weeks)  Median (IQR) | 30.0 (29.4 – 31.4) | 30.1 (28.0-31.6) |
| **BMI** (kg/m^2^)  Median (range) | 28 (25-31) | 27 (25-35) |
| **Parity** no. (%) *  0  1  ≥2 | 5 (29)  8 (47)  4 (23) | 38 (72)  10 (19)  5 (9) |
| **SBP at Booking** (mmHg)  Median (IQR) | 120 (118-120) | 120 (110-125) |
| **SBP at Delivery** (mmHg)  Median (IQR) **** | 120 (112-130) | 175 (160-182) |
| **DBP at Delivery** (mmHg)  Median (IQR) **** | 70 (65-80) | 100 (95-110) |
| **Birth weight** (g) *  Median (IQR) | 1451 (1237-1790) | 1146 (870-1426) |
| **Highest protein creatinine ratio (g/mmoL) §**  Median (IQR) | -- | 0.25 (0.13-0.71) |
| **Liver function abnormalities**  Number (%) | -- | 33 (62) |
| **Thrombocytopenia <150 (x10^9/L)**  Number (%) | -- | 17 (32) |

BMI data available for 12/17 preterm controls and 49/53 preeclamptic women. SBP at booking available for 8/17 preterm controls and 16/53 preeclamptic women.*p<0.05 ****p<0.0001 § Normal range <0.03

**Supplementary table 5: Demographics of patients with established, severe early onset preeclampsia, protein placental tissue samples.** There was no significant difference seen in maternal age, gestation at delivery or BMI.

Supplementary Figure 1

**Supplementary Figure 1: *GATA2* is unchanged in peripheral blood monocytes (PBMCs) and granulocytes exposed to cytotrophoblast conditioned media.** To assess whether white blood cell *GATA2* expression is modulated by placental factors, isolated white blood cells and platelets were exposed to cytotrophoblast conditioned media, however no significant change in white blood cell *GATA2* expression was observed (p=0.85). Experiments were repeated n=5 times, with each ‘n’ representing a separate primary PBMC/granulocyte isolation and including experimental triplicates. *p<0.05, **p<0.01, ***p<0.001, ****p<0.0001. (Data displayed as median with interquartile range).

Supplementary Figure 2


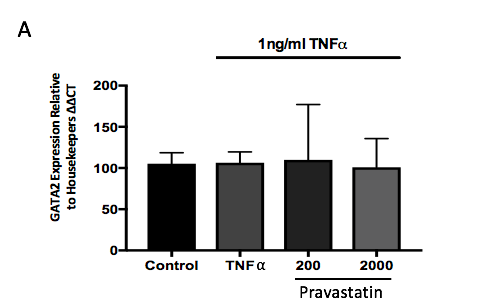


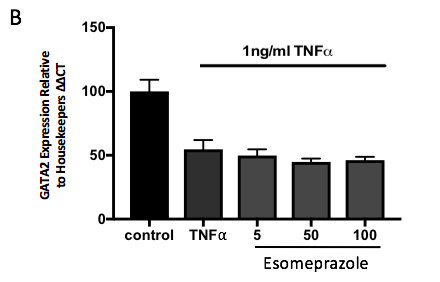


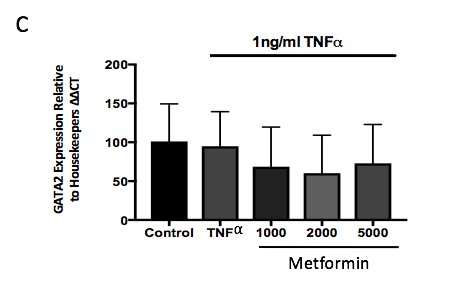


**Supplementary Figure 2: Pravastatin, esomeprazole and metformin do not increase *GATA2* mRNA expression in primary HUVECs.** We have previously reported that pravastatin, esomeprazole and metformin improve endothelial dysfunction *in vitro*. To assess whether *GATA2* is altered by pravastatin, esomeprazole or metformin, primary HUVECs were treated with TNFα in the presence of increasing doses of the drugs. **A,** *GATA2* mRNA expression is not significantly altered when HUVECS are treated with **A,** pravastatin (data displayed as median with interquartile range); **B,** esomeprazole, (data displayed as mean with standard error of the mean); or **C,** metformin (data displayed as median with interquartile range). Experiments were repeated a minimum of n=3 times, with each ‘n’ representing a separate primary HUVEC isolation and including experimental triplicates. *p<0.05, **p<0.01, ***p<0.001, ****p<0.0001

Supplementary figure 3
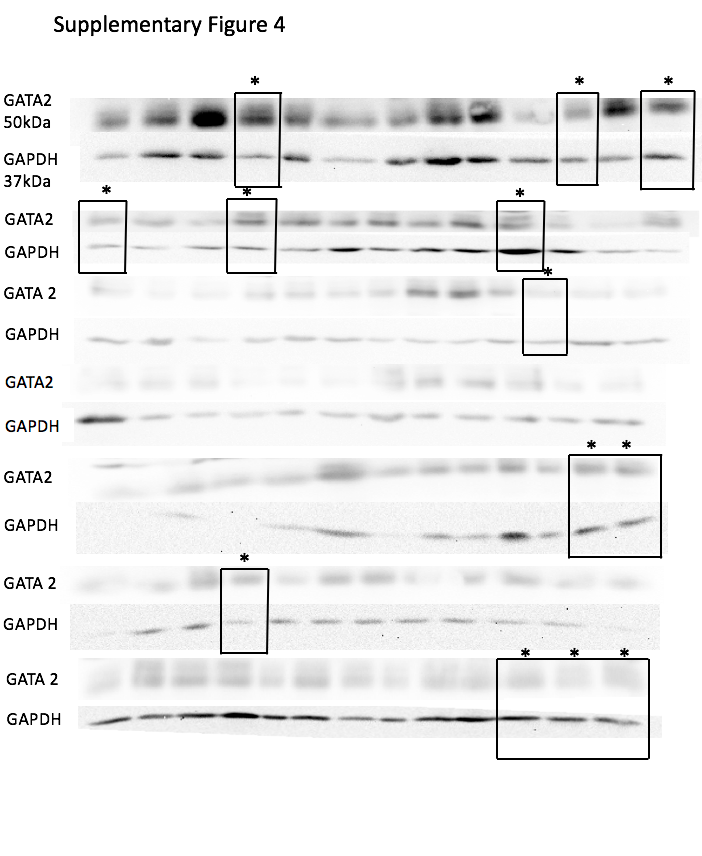
**Supplementary Figure 4: Western Blot Gels.** Full western blots of samples that were analysed for expression of GATA2 in placental tissues (shown in Figure 2). After recharacterisation of patient samples several were excluded, indicated with ‘*’. Gel 1 position 4 excluded as does not meet the definition of preeclampsia as per described in methods. Gel 1 position 11 excluded as vaginal delivery not LUSCS. Gel 1 position 13 excluded due to chorioamnionitis. Gel 2 position 1 excluded as vaginal delivery. Gel 2 position 4 excluded due to severe IUGR. Gel 2 position 10 due to type 1 diabetes mellitus, severe IUGR and renal disease. Gel 3 position 11 excluded due to chorioamnionitis diagnosis. Gel 5 position 11 excluded due to severe IUGR. Gel 5 position 12 excluded due to preexisting hypertension. Gel 6 position 4 excluded due to severe IUGR. Gel 7 position 11 excluded due to chorioamnionitis. Gel 7 position 12 excluded due to vaginal delivery. Gel 7 position 13 excluded as was laboring with an abnormal CTG.
